# Association of Statins With Functional Outcome and 30-Day Mortality in Patients With Intracerebral Hemorrhage

**DOI:** 10.7759/cureus.14421

**Published:** 2021-04-11

**Authors:** Joana Silva Marques, Giovana Ennis, Gabriela Venade, Rita João Soares, Nuno Monteiro, Ana Gomes

**Affiliations:** 1 Internal Medicine, Centro Hospitalar Tondela-Viseu, Viseu, PRT; 2 Stroke Unit, Centro Hospitalar Tondela-Viseu, Viseu, PRT

**Keywords:** intracerebral hemorrhage, hemorrhagic stroke, statins, mortality, modified rankin score

## Abstract

Aim

The effect of statins is well established in cardiac and cerebrovascular diseases. However, its impact on intracerebral hemorrhage (ICH) is unclear. We aim to identify an association of pre-ICH statin treatment and statin use during admission for ICH with functional outcome at discharge and 30-day mortality.

Material and methods

A retrospective cohort study was held in patients with ICH admitted to our stroke unit over a year period. Demographic characteristics, risk factors and cardiovascular diseases, Glasgow Coma Scale (GCS), National Institutes of Health Stroke Score (NIHSS), systolic blood pressure (SBP) at admission, cholesterol levels and radiologic findings were analyzed to explore the association between pre-ICH and inpatient statin use with outcomes. The primary endpoint was functional outcome defined as modified Rankin Score (mRS) at discharge and 30-day mortality. We performed a univariate analysis and the variables with statistical significance were included in a multivariate analysis to control for confounding covariates.

Results

The study included 78 patients, 33 (42.31%) had previous statin intake history, of which 13 (39.39%) maintained statin intake during hospitalization. Regarding functional outcome we did not report a statistically significant difference between groups. In the “pre-ICH statin use” group a decreased 30-day mortality (6.06%, p = 0.009) was observed. In this group it was also noted higher antiplatelet medication use (33.33%, p = 0.006), higher GCS at admission (13-15: 84.38%, p = 0.018) and deep ICH (81.82%, p = 0.030). However, 30-day mortality had no impact in multivariate regression (Odds ratio (OR) 4.535, 95% Confidence Interval (CI) = 0.786-26.173, p = 0.091). In the group that maintained statin treatment during hospitalization no deaths were registered (p = 0.020) and there was no association with functional status. Multivariate regression analysis was not performed due to sample size.

Conclusion

The only association demonstrated in this study was lower 30-day mortality with pre-ICH statin use and continued statin treatment during admission. However, this was not confirmed by multivariate regression analysis. There were no differences between groups concerning cholesterol values, results that can be explained by the pleiotropic and immunomodulatory effect of statins. However, prospective studies are needed to prove the benefit of the statins in ICH.

## Introduction

Intracerebral hemorrhage (ICH) is the second most common cause of stroke (10-15% of all strokes) [1]. The 30-day mortality ranges from 35% to 52% and half of these deaths occur within the first two days [2]. Given the morbidity and mortality associated with ICH, assessment of therapies with neuroprotective effects is of increasing interest.

Statins inhibit, in a reversible way, the HMG-CoA reductase, which is the limiting step in cholesterol synthesis [3]. This mechanism is responsible for dyslipidemia treatment and the reduction of primary and secondary cardiovascular events [3]. Despite these benefits, a post hoc analysis from Stroke Prevention by Aggressive Reduction in Cholesterol Levels (SPARCL) trial showed a three-fold increase of recurrent ICH in patients treated with statin [4]. There are data regarding the contribution of low levels of serum cholesterol to the cerebrovascular endothelium fragility and statin treatment to the decreased platelet aggregation and thrombogenesis. Both mechanisms could promote hematoma expansion, increase the risk for recurrent hemorrhage, and aggravate ICH mortality or functional outcomes [5-6].

Statins also have pleotropic effects, like anti-inflammatory, antithrombotic, antioxidative, and neuroprotective. These are being demonstrated in animal and human models [3]. In this regard, a few studies reported that statin use was associated with reduced mortality and improved functional outcome after ICH. There is a paucity of data regarding beneficial impact of stating in acute ICH.

Therefore, we set out to investigate the association between pre-ICH statin treatment and statin use during admission with functional outcomes and 30-day mortality.

This article was previously presented as a meeting poster at the 26th National Congress of Internal Medicine of the Portuguese Society of Internal Medicine on August 27, 2020.

## Materials and methods

We performed a retrospective study on primary ICH patients admitted in a single-center stroke unit, from 1st of June 2018 to the 31st of May 2019. All patients with a baseline admission computerized tomography (CT) scan and medical records of pre-ICH statin use were eligible. Individuals with no inclusion criteria, diagnosed with subdural or subarachnoid hemorrhage, ischemic stroke with hemorrhagic transformation, traumatic hemorrhage, bleeding cerebral tumor and cerebral vascular malformation were excluded.

Clinical information, which included chronic medication, was obtained from patients during the medical interview and complemented by medical records. Medical record review data included: pre-ICH statin use, statin treatment during hospitalization, age, gender, prior history of hypertension, diabetes mellitus (DM), cerebral vascular disease (CVD), body mass index (BMI), previous antiplatelet or anticoagulant treatment. Glasgow Coma Scale (GCS) and National Institutes of Health Stroke Score (NIHSS) at admission were considered and categorized according to cut-off points (GCS: 4-8, 9-12, 13-15; NIHSS: 0-4, 5-8, 9-16, 16-24) and CT characteristics were pointed out like hematoma locations, presence of intra-ventricular hematoma extension and volume. ICH location was categorized into lobar, deep, cerebelous and brainstem and volume calculated by A · B · C/ 2 method. Total cholesterol, low density lipoprotein (LDL), high density lipoprotein (HDL) and triglycerides level were also recorded.

Statin use was further dichotomized into prior-ICH use and no use and continued or discontinued during hospitalization.

The primary endpoints were the effect of statin use on functional status at discharge and 30-day mortality in ICH patients. Functional status was defined by modified Rankin Score (mRS) considering good outcome 0-2 and bad outcome 3-6.

Statistical analysis was performed in two steps. First, a univariate analysis was done. Statistically significant results were used in a multivariate regression analysis to explore the effect of pre-ICH statin use and statin continuation during hospitalization in functional outcome at discharge and 30-day mortality.

Categorical variables are presented as frequencies and percentages, and continuous variables as median and interquartile range due to skewed distributions or mean and standard deviation. Normal distribution was checked using Shapiro-Wilk test. Categorical variables were compared with chi-square test and continuous variables were compared with Mann-Whitney or t-student in non- or parametric test respectively. All reported “p” values are two-tailed, with a “p” value of 0.05 indicating statistical significance (CI 95%). Analyses were performed with the use of “Statistical Package for the Social Sciences” (SPSS) version 26 (IBM Corp., Armonk, NY).

## Results

Eighty-three ICH patients were admitted in the stroke unit over the study period. Five cases were excluded due to missing data about pre-ICH statin use.

Our final study population included 78 patients, of which 57.70% (45) were male. Median age was 76.00+/-14.00 years, a median length of stay in the stroke unit was 4.24+/-2.35 days and 17.35+/-20.74 days in other medical wards. Overall, 11 patients (14.10%) had good functional outcome and 16 (20.51%) patients died during the first 30 days after ICH, of which 10 (62.50%) occurred in stroke unit and six (37.50%) in other wards.

Thirty-three (43.31%) had medical history of statin use and 45 (57.69%) had no pre-ICH statin use. In the statin user group, 13 (39.40%) continued statins and 20 (60.61%) discontinued statin during admission.

Table 1 compares the baseline characteristics and outcomes of interest among statin and non-statin users, respectively. The univariate analysis showed with statistical significance that “pre-ICH statin” group was associated to a higher number of patients with antiplatelets use (33.33%, p = 0.006), higher GCS at admission (13-15: 84.38%, p = 0.018) and deep hemorrhage, with no cases of cerebellum or brainstem hemorrhage (81.82%, p = 0.030) (Table 1). Although no statistical significance was found, in this group the patients were older (78.00+/-11.50, p = 0.451), had more hypertension (78.10%, p = 0.087) and CVD (36.36%, p = 0.060) and higher BMI (29.40+/-6.06, p = 0.50). This same group was associated with, NIHSS at admission 0-4 and 9-16 (25.00%, 45.00%, respectively, p = 0.189), lower systolic blood pressure (SBP) (166.42+/-61.57, p = 0.778) smaller hemorrhage volume on head CT (12.80+/-57.20, p = 0.295) and fewer cases of intra-ventricular hematoma (42.42%, p = 0.819). The “pre-ICH statin” group had better cholesterol levels with lower total cholesterol (TC) (153.00+/-43.00, p = 0.286), lower low density lipoprotein (LDL) (85.40+/-35.10, p = 0.58), lower triglycerides (TG) (105.35+/-47.93, p = 0.582) and higher high density lipoprotein (HDL) (46.10+/-10.50, p = 0.286).

**Table 1:** Univariate analysis of different variables and association with pre-ICH statin use BMI: Body Mass Index; CVD: Cerebral Vascular Disease; DM: Diabetes mellitus; GCS: Glasgow Coma Scale; HDL: High Density Lipoprotein; HT: Hypertension; ICH: Intracerebral hemorrhage; LDL: Low Density Lipoprotein; mRS: modified Rankin Score; NIHSS: NIH Stroke Scale; OAC: Oral Anticoagulants; SBP: Systolic Blood Pressure; TC: Total Cholesterol; TG: Triglycerides.

	Total number cases, n = 78	Pre-ICH statin, n = 33	No pre-ICH statin, n = 45	p
Age, years	76.00+/-14.00	78.00+/-11.50	76.00+/-18.00	0.451
Gender, male	45 (57.69)	18 (54.55)	27 (60.00)	0.650
HT	51 (66.20)	25 (78.0)	26 (57.80)	0.087
DM	33 (42.31)	14 (42.42)	19 (42.22)	1.000
CVD	19 (24.36)	12 (36.36)	7 (15.56)	0.060
BMI, kg/m²	27.24+/-4.53	29.40+/-6.06	26.42+/-5.66	0.50
OAC	14 (17.95)	8 (24.24)	6 (13.33)	0.244
Antiplatelets	14 (17.95)	11 (33.33)	3 (6.67)	0.006
TC (mg/dL)	161.00+/-47.00	153.00+/-43.00	169.50+/-73.00	0.286
LDL (mg/dL)	93.15+/-37.08	85.40+/-35.10	102.50+/-45.35	0.58
HDL (mg/dL)	44.80+/-11.90	46.10+/-10.50	40.00+/-15.90	0.353
TG (mg/dL)	101.80+/-61.50	105.35+/-47.93	107.25+/-80.85	0.582
NIHSS admission				0.189
0-4	7 (17.50)	5 (25.00)	2 (10.00)	
5-8	9 (22.50)	4 (20.00)	5 (25.00)	
9-16	15 (37.50)	9 (45.00)	6 (30.00)	
17-24	9 (22.50)	2 (10.00)	7 (35.00)	
GCS admission				0.018
4-8	5 (6.49)	1 (3.13)	4 (8.89)	
9-12	21 (27.27)	4 (12.50)	17 (37.78)	
13-15	51 (66.23)	27 (84.38)	24 (53.33)	
SBP (mmHg)	167.68+/-32.22	166.42+/-61.57	168.58+/-33.02	0.778
ICH location				0.030
Lobar	23 (29.49)	6 (18.18)	17 (37.78)	
Deep	50 (64.10)	27 (81.82)	23 (51.11)	
Cerebellum	3 (3.75)	0 (0)	3 (6.67)	
Brainstem	2 (2.56)	0 (0)	2 (4.44)	
Volume (mm^3^)	21.00+/-52.30	12.80+/-57.20	24.00+/-48.30	0.295
Intra-ventricular hematoma	35 (44.87)	14 (42.42)	21 (46.67)	0.819
mRS discharge				0.176
0-2	11 (14.10)	7 (21.21)	4 (8.89)	
3-6	34 (43.59)	13 (39.40)	21 (46.67)	
30-day mortality	16 (20.51)	2 (6.06)	14 (31.11)	0.009

Comparing with primary outcomes there was no significant differences between groups in functional outcome at discharge (p = 0.176) but a lower mortality was observed in the “pre-ICH statin” group with statistical significance (6.06% vs 31.11%, p = 0.009) (Table 1). In multivariate analysis (Table 2), the group of pre-ICH statin use did not show any significant impact in 30-day mortality after adjusting to other variables (OR 4.535, 95% CI 0.786-26.173, p = 0.091).

**Table 2:** Multivariate analysis of pre-ICH use and 30-day mortality CI: Confidence interval; GCS: Glasgow Coma Score; ICH: Intracerebral Hemorrhage; OR: Odds Ratio

	OR	p	CI
Antiplatelets	0.850	0.873	0.117-6.168
GCS	5.830	0.001	2.005-16.949
ICH location	1.023	0.963	0.391-2.679
Pre-ICH statin	4.535	0.091	0.786-26.173

A significant association between fewer CVD history (23.08%, p = 0.038), more antiplatelets use (38.46%, p = 0.008), lower NIHSS at admission (0-4: 30.77% and 9-16: 30.77%, p = 0.05) and higher GCS (13-15: 100%, p = 0.033) was observed in the continued statin therapy during admission group (Table 3). Regarding functional outcome we did not register statistical differences between groups (p = 0.282). No deaths were observed in the group that continued statin therapy during admission and only two in the group that discontinued therapy. This data did not fit our previous model.

**Table 3:** Univariate analysis of different variables and association with continued statin treatment during admission BMI: Body Mass Index; CVD: Cerebral Vascular Disease; DM: Diabetes mellitus; GCS: Glasgow Coma Scale; HDL: High Density Lipoprotein; HT: Hypertension; ICH: Intracerebral Hemorrhage; LDL: Low Density Lipoprotein; mRS: modified Rankin Score; NIHSS: NIH Stroke Scale; OAC: Oral Anticoagulants; SBP: Systolic Blood Pressure; TC: Total Cholesterol; TG: Triglycerides.

	Pre-ICH statin, n = 33	Continued statin therapy during admission, n = 13	Discontinued statin therapy during admission, n = 20	p
Age, years	78.00+/-11.50	79.00+/-14.50	77.50+/-9.50	0.748
Gender, male	18 (54.55)	7 (53.84)	11 (55.00)	0.889
HT	51 (66.20)	10 (76.92)	15 (75.00)	0.158
DM	33 (42.31)	5 (38.46)	9 (45.00)	0.933
CVD	19 (24.36)	3 (23.08)	9 (45.00)	0.038
BMI, kg/m²	27.24+/-4.53	29.40+/-8.09	28.96+/-6.40	0.141
OCA	14 (17.95)	3 (23.08)	5 (25.00)	0.459
Antiplatelets	14 (17.95)	5 (38.46)	6 (30.00)	0.008
TC (mg/dL)	161.0+/-47.00	158.00+/-91.75	151.00+/-42.00	0.356
LDL (mg/dL)	93.15+/-37.08	93.00+/-82.37	85.75+/-37.85	0.105
HDL (mg/dL)	44.80+/-11.90	44.85+/-10.88	46.75+/-10.40	0.640
TG (mg/dL)	101.80+/-61.50	112.10+/-41.45	105.35+/-59.70	0.801
NIHSS admission				0.05
0-4	7 (17.50)	4 (30.77)	1 (5.00)	
5-8	9 (22.50)	0 (0)	4 (20.00)	
9-16	15 (37.50)	4 (30.77)	5 (25.00)	
17-24	9 (22.50)	0 (0)	2 (10.00)	
GCS admission				0.033
4-8	5 (6.49)	0 (0)	1 (5.00)	
9-12	21 (27.27)	0 (0)	4 (20.00)	
13-15	51 (66.23)	13 (100.00)	14 (70.00)	
SBP (mmHg)	167.68+/-32.22	169.00+/-25.81	164.56+/-35.77	0.706
ICH location				0.163
Lobar	23 (29.49)	3 (23.08)	3 (15.00)	
Deep	50 (64.10)	10 (76.92)	17 (85.00)	
Cerebellum	3 (3.75)	0 (0)	0 (0)	
Brainstem	2 (2.56)	0 (0)	0 (0)	
Volume (mm^3^)	21.00+/-52.30	16.05+/-50.45	14.55+/-53.83	0.343
Intra-ventricular hematoma	35 (44.87)	2 (15.38)	12 (60.00)	0.039
30-day mortality	16 (20.51)	0 (0)	2 (10.00)	0.020
mRS discharge				0.282
0-2	11 (14.10)	3 (23.08)	4 (20.00)	
3-6	34 (43.59)	4 (30.77)	9 (45.00)	

## Discussion

In our population, pre-ICH statin treatment and statin use during hospitalization was not associated with functional outcomes at discharge. A trend associating statin use pre-ICH or during admission with decreased 30-day mortality was noted with statistical significance but with no impact after adjusting for other potential confounders.

The findings of our study shed light on the contradictory data about the influence of statins on outcomes after ICH. This is a complex disease and involves several factors that independently or co-dependently affect the outcome of these patients.

In 2004, SPARCL trial found out that treatment with atorvastatin was independently associated with an increased risk of hemorrhagic stroke (hazard ratio, 1.68; 95% CI, 1.09-2.59) [4]. However, this trial enrolled patients with prior ischemic stroke and with probable secondary microvascular injury [3].

There are studies that reported increased rates of ICH and ICH-related mortality in patients with low cholesterol levels [7] and that low LDL was an important predictor of hematoma growth [6]. It has been hypothesized that cholesterol may be important for cerebrovascular wall integrity and that low levels may increase the risk of vessel rupture and higher fibrinolytic activity [8]. Statins are lipid-lowering therapy so we should expect a bad outcome in patients with pre-ICH statin use [7].

In our study, the results were independent of cholesterol levels. It was also noted an association between pre-ICH statin use and better GCS at admission. Hence, the clinical benefit may not be limited to the lipid-lowering properties of statins but also derived from other “pleiotropic” effects [7]. In animal experiments it has been possible to demonstrate the capacity of statins to improve ICH mortality by the promotion of neuronal plasticity and the limitation of damage in the boundary tissue [9], the increase of cerebral perfusion (mediated via increased endothelial nitric oxide secretion resulting in vasodilatation), the anti-inflammatory proprieties, the improvement of angiogenesis and neurogenesis after ischemic injury. There is also evidence that statin discontinuation could lead to a rebound effect resulting in oxidative stress and vascular disfunction [10].

Supporting these findings, many observational studies in humans were performed, but with contradictory findings (Table 4). In 2007, Naval et al.'s study was the first retrospective study that reported the benefit of pre-ICH statins treatment in humans by reducing early absolute edema volume and decreasing 30-day mortality (OR 0.080, p = 0.050), with no effect in functional outcomes at discharge [11]. FitzMaurice et al. found no effect of pre-ICH statin use on the rates of functional independence (28% versus 29%, P 0.84) or mortality (46% versus 45%, P 0.93) and ICH survivors treated with statins after discharge did not have a higher risk of recurrence (adjusted HR 0.82, 95% CI 0.34-1.99, P 0.66) [11]. However cholesterol and triglycerides levels were not reported in any group, and medical comorbidity was more common in statin pretreated patients than in the non-pretreated group. Eichel et al. found that despite having significantly smaller haematomas, patients that were using statins did not have better neurological or functional outcomes at 90-day post-ICH [12]. Statin users more often had existing co-morbidities that adversely affect outcomes. This data points to the fact that statin users represent a generally more impaired population, and that these comorbidities can counteract the possible beneficial effects of statin use. Flint et al. designed a multicentric retrospective study and reported an increased mortality in the group that discontinued statin during hospitalization. Multivariable regression showed a relationship between statin use and higher probability of 30-day survival (0.15 [95% CI, 0.04-0.25]; P = .01). Winkler et al. had a longer follow-up (12 months) and concluded that pre-ICH statin group was associated with decreased mortality in-hospital and 12 months (p = 0.001) confirmed after multivariable adjustments [13].

**Table 4:** Clinical studies investigating the effects of statins on ICH AVM: Arteriovenous malformation; CT: Computerized Tomography; CI: Confidence Interval; CNS: Canadian Neurological Scale; GOS: Glasgow Outcome Scale; ICH: Intracerebral Hemorrhage; IQR: Interquartile Range; IVH: Intraventricular Hemorrhage; KPNC: Kaiser Permanente Northern California; mRS: modified Rankin Score; OR: Odds Ratio; SAH: Subarachnoid hemorrhage; SDH: Subdural hemorrhage.

	Study type	Cohort	Exclusions	End-points	Results
Naval et al., 2007 [11]	Retrospective	125, 26% statin	Trauma, cerebral tumour, aneurisms, AVM, infratentorial ICH	30-day mortality	Multiple logistic regression analysis, prior statin use (P = 0.05) was found to be associated with decreased mortality with a greater than 12-fold odds of survival
FitzMaurice et al., 2008 [12]	Retrospective	629, 24% statin	Secondary ICH, no 90-day (GOS) 90 days	Good 90-day outcome (GOS 4-5)	No effect of pre-ICH statin use on the rates of functional independence (28% versus 29%, P 0.84) or mortality (46% versus 45%, P 0.93). Multivariable analysis for independent status in pre-ICH statin users was 1.16 (95% CI 0.65 to 2.10, P 0.62). ICH survivors treated with statins after discharge did not have a higher risk of recurrence (adjusted HR 0.82, 95% CI 0.34 to 1.99, P 0.66).
Leker et al., 2009 [14]	Retrospective	312, 28,5% statin	Secondary ICH	Good functional outcome at discharge (mRS 0-3). Discharge at nursery facility, home or death	Pre-ICH statin had higher proportions of mRS 0-3, lower death rates, and higher rates of discharge home or to a rehabilitation facility. Logistic regression analyses of pre-ICH statin OR 2.97 for mRS 0-3 (95% CI; 1.25 to 7.35) at discharge and OR 0.25 for death or nursing facility disposition (95% CI; 0.09 to 0.63).
Eichel et al., 2010 [13]	Retrospective	399, 25,3% statin	Secondary ICH (hemorrhagic transformation from stroke, tumor, AVM), SAH, SDH	90-day mortality. Good 90-day functional outcome (mRS < 2)	Pre-ICH statin had no association with primary endpoints mRS < 2 or mortality. No impact on multiple logistic regression analysis.
Gomis et al., 2010 [15]	Retrospective	269, 12.6% statin	Secondary ICH, ICH due to anticoagulation therapy, primary IVH, mRS basal >1	Good 90-day functional outcome (mRS 0-2)	Multivariate regression analysis showed a significant association between age (OR: 0.95; CI 0.92–0.97), ICH volume (OR: 0.96; CI 0.94–0.98), GCS (OR: 1.55; CI 1.21–1.98), pre-treatment with statins (OR: 4.21; CI 1.47–12.17; P = 0.008), and mRS 0-2 at 3 months.
Biffi et al., 2011 [1]	Retrospective	699, 34.0% statin	Secondary ICH	Good 90-day functional outcome (mRS 0-2) 90-day mortality	Association between statin use before ICH and increased probability of favorable outcome (OR 2.08, 95% CI 1.37– 3.17). Reduced mortality (OR 0.47, 95% CI 0.32–0.70) at 90 days. Meta-analysis of all published evidence confirmed the effect of statin use on good outcome (OR 1.91, 95% CI 1.38–2.65) and mortality (OR 0.55, 95% CI 0.42–0.72) after ICH
Romero et al., 2011 [16]	Prospective	83, 24% statin	Secondary ICH, No CT at admission, no important data	90-day outcome (GOS) 90-day mortality	No effect from pre-ICH statin use on the functional independence rates (32% vs 36%, P = 0.79) or mortality (41% versus 47%, P = 0.82).
Dowlatshahi et al., 2012 [17]	Retrospective	2466, 21.8% statin	Secondary ICH	Bad outcome at discharge (mRS 4-6) 30-day and 180-day mortality	No association with primary outcomes, Statins were discontinued on admission, had poor outcome (90% vs 62%, P 0.01), and higher 30-day mortality (71% vs 21%, P 0.01). After adjusting for stroke severity, statin discontinuation was still associated with poor outcome (adjusted OR, 2.4; 95% CI, 1.13–4.56) and higher mortality (adjusted OR, 2.0; 95% CI, 1.30–3.04).
King et al., 2012 [18]	Prospective	1381, 21.1% statin	Trauma, Secondary ICH, stroke, tumor, AVM, ICH due to anticoagulant therapy, no data regarding previous medication	30-day mortality	Multivariate logistic regression did not demonstrate any effect of prior statin use (p = 0.781) on mortality.
Mustanoja et al., 2013 [19]	Retrospective	964, 19% statin		Good functional outcome (mRS ≤ 2). Hospitalization, 30-day and 90-day mortality	mRS at discharge or mortality did not differ between groups (pre-ICH statin use). Compared with survivors, significantly lower total cholesterol and low-density lipoprotein cholesterol levels were observed in patients who died in hospital (median, 4.1 mmol/L [IQR, 3.6–4.4] versus 4.5 [3.8–5.1).
Flint et al., 2014 [20]	Retrospective	3481, 34.3% statin	Previous ICH, not living KPNC range, no information regarding statin use	30-day mortality. Discharge to home or inpatient rehabilitation facility	Inpatient statin users were more likely than nonusers to be alive 30 days after ICH (OR 4.25 [95% CI, 3.46-5.23]; P < .001) and were more likely to be discharged to their home or an acute rehabilitation facility (OR, 2.57 [95% CI, 2.16-3.06]; P < .001). Statin therapy was discontinued, nonusers were less likely than statin users to survive to 30 days (OR, 0.16 [95% CI, 0.12-0.21]; P < .001) and were less likely than statin users to be discharged to their home or an acute rehabilitation facility (OR, 0.26 [95% CI, 0.20-0.35]; P < .001). Multivariable regression showed that statin therapy was associated with a higher probability of 30-day survival (with an increase in probability of 0.15 [95% CI, 0.04-0.25]; P = .01) and a better chance of being discharged to home or an acute rehabilitation facility (with an increase in probability of 0.13 [95% CI, 0.02-0.24]; P = .02)
Winkler et al., 2013 [21]	Retrospective	426, 44,6% statin	Secondary ICH	Hospitalization and 12-month mortality. 12-month blood functional outcome (Barthel index)	Pre-ICH statin group was associated with decreased mortality in-hospital and 12 months (p = 0.001). Multivariable analysis found a decreased odds of death or disability at 12 months after ICH (OR 0.44; 95% CI 0.21-0.95).
Siddiqui et al., 2017 [22]	Retrospective	2457, 10.9% statin	Secondary ICH, no data regarding functional status or hematoma volume	90-day mortality. Bad 90-day functional outcome (mRS)	Statin use was associated with reduced mortality and disability without any effect on hematoma growth. This association was primarily driven by continued/new statin use. Multivariate analysis showed continued/new statins users had good outcomes over prior users. However, statins may have been continued/started more frequently among less severe patients. Propensity score was developed based on factors that could influence a physician’s decision in prescribing statins and used as a covariate, continued/new statin use was no longer a significant predictor of good outcome.

These contradictory studies could be explained by heterogeneity of the sample size (from 83 to 3481 patients) and outcomes. Only two prospective studies were performed, and both did not observe any association between pre-ICH statin use and 30-day mortality.

A few limitations to our study should be pointed out. This is an observational retrospective study although data was collected prospectively. It is possible that unmeasured or unknown confounders may influence the results. However, multiple regression helped to reduce this potential bias. We had a small sample size which could decrease the power of our study and make it difficult to test whether any particular statin or dose had superior effects. There was a lack of information regarding some variables like BMI (n = 34), lipidic profile at admission (n = 43-46), NIHSS at admission (n = 40) and mRS at discharge (n = 45). The later was a limitation to investigate the association between pre-ICH statin use and continuation during hospitalization and functional outcome at discharge. Also, clinical outcomes (mRS and mortality) were evaluated at discharge and not in a prolonged subacute time frame to maximize clinical recovery after ICH. Nevertheless, we acknowledge that neurological recovery following stroke may take place over longer periods of time and the variability in the time period following ICH that these patients were discharged might affect the data assessing functional outcomes.

## Conclusions

In our study, pre-ICH statin use or continued treatment during admission was not associated with improved functional status. However, an association between these groups and decreased 30-day mortality was reported. These findings did not persist after adjusting for other variables.

Preclinical and clinical studies support the potential neuroprotective and recovery enhancement effects afforded by statins in the setting of acute ICH. There are conflicting data influenced due to heterogeneity of retrospective studies and absence of large prospective trials evaluating the safety and efficacy of statin therapy in ICH. Considering that the interpretations of our results are restricted by several limitations, future studies considering the impact of statins on mortality and functional outcomes are needed.
